# Cutaneous Manifestation of Diabetes Mellitus in an Unsheltered Male Leading to Emergent Hospitalization

**DOI:** 10.7759/cureus.22432

**Published:** 2022-02-21

**Authors:** Taha F Rasul, Alana Moore, Daniel R Bergholz, Kavan Mulloy, Armen Henderson

**Affiliations:** 1 Department of Medical Education, University of Miami Miller School of Medicine, Miami, USA; 2 Department of Allergy and Immunology, University of Miami Miller School of Medicine, Miami, USA; 3 Department of Internal Medicine, University of Miami Hospital, Miami, USA

**Keywords:** lower limb dermatology, homelessness, health care literacy, outreach programs, diabetic lower extremity ulcer, type 1 diabetes mellitus (t1dm), street medicine

## Abstract

Diabetes mellitus (DM) is one of the most common chronic diseases in the United States. It is characterized by increased patient morbidity and mortality due to the many complications that can arise. Certain dermatological findings can be indicative of poorly controlled DM and can be a useful clue to further management. Persons experiencing homelessness (PEH) with DM often have higher rates of diabetic complications than the general diabetic population. Medical providers caring for PEH in the setting of limited resources should carefully evaluate cutaneous disease as a potential indicator of underlying illness. This physical manifestation of illness can serve to guide the next appropriate steps in management. A 41-year-old unsheltered male with an extensive medical history of hypertension, seizures, chronic diarrhea, and cocaine use was seen at a “foot-washing” medical outreach event. He presented with fevers, chills, and multiple painless right lower extremity ulcerated lesions of unspecified origin. A finger-stick glucose measurement was found to be 650 mg/dL. After immediate administration of 10 units of insulin, he was transported immediately to the emergency department and admitted. His month-long hospital course was complicated and involved the amputation of multiple toes. Preemptive outreach and management could have prevented the marked deterioration of his disease and represents the importance of outreach and regular follow-up with the PEH community.

## Introduction

Diabetes mellitus (DM) is the most expensive chronic disease in the nation, costing approximately US$327 billion in 2017 [1]. Despite its high cost of treatment, there is insufficient literature regarding the prevalence, management, and outcomes of DM in chronically unsheltered patient populations. DM has a wide spectrum of presentations and affects virtually every major organ system [2]. The integumentary system is no exception to this, and cutaneous manifestations of DM can sometimes be the first sign of the disease. Individuals with type 2 DM (T2DM) have a higher likelihood of skin manifestations than those with type 1 DM (T1DM) [3]. Some of the most feared yet common cutaneous complications are infections. These can occur from pre-existing skin lesions or can occur hematogenously due to impaired immune responses in diabetic patients [4].

Individuals diagnosed with DM on average have medical expenses of US$16,752, more than half of which is due to diabetic management. This figure is approximately 2.3 times higher than the average expense for patients without DM [5]. The uninsured population is seen to have 168% more emergency department (ED) visits than their insured counterparts. A study conducted to explore this discrepancy in ED utilization among cohorts of DM showed within their unadjusted data that within a year timeframe, 26% of unstably housed participants reported a DM-related ED visit compared to 7% of those who were stably housed for that year [6]. Multiple studies have also shown homelessness in general to be associated with substandard DM management [7]. This can exacerbate their underlying predisposition toward chronic wounds, infections, and cardiovascular disease.

A 2018 Medicare analysis identified that roughly eight million people in the United States had wounds, both infectious and noninfectious [8]. These figures did not include the disproportionate prevalence of wounds in homeless individuals. Some of the highest expenses in terms of wound care are for surgical wounds. The second-highest price tag is for diabetic foot ulcers, likely due to the chronicity and recurrence of such wounds [9]. Although current research is being guided toward the mechanistic research of cutaneous wound complications, there is a sparsity of literature on the incidence and prevalence of both acute and chronic wounds in the homeless populations in the United States. Estimates of the true number of homeless individuals in the United States have not been updated to accurately reflect the dramatic increase in homelessness due to the coronavirus pandemic and individuals with refugee or undocumented status. Even with annual nationwide surveys, it is likely that the Department of Housing and Urban Development’s current numbers for homelessness (approximately 580,000) are underreported [10]. It has been well established that homeless individuals suffer from a disproportionate burden of disease. This is why, on average, the life expectancy of an unsheltered homeless person is 55 years, which is more than 20 years below the national average [11]. There is not enough information about the DM status of homeless individuals, which is especially important because management of diabetic complications is one of the main reasons for ED visits. In fact, patients admitted with recurrent episodes of diabetic ketoacidosis (DKA) had significantly higher rates of depression, alcohol and drug use, and homelessness [12]. This represents a public health crisis because complications from DM are not only limited to DKA but also the management of chronic wounds and dermatologic conditions. Chronic ulcers, particularly those seen in long-standing, poorly controlled DM have an increased likelihood of infection. Potential downstream complications of these wounds can include cellulitis, sepsis, and osteomyelitis.

The early detection and management of chronic diseases in such a vulnerable population can therefore decrease the likelihood of life-threatening complications from occurring and potentially decrease ED utilization. We present a case of uncontrolled T1DM in an unsheltered patient, which was discovered incidentally through cutaneous findings at a public health outreach event.

## Case presentation

The Miami Street Medicine organization attempted a volunteer-driven approach to the management of foot ulcers and wound complications in high-risk, low socioeconomic status patients. A “foot-washing” event was conducted as a means to tend to and assess the overall health and prevalence of foot conditions in patients belonging to the impoverished Overtown neighborhood.

**Figure 1:**
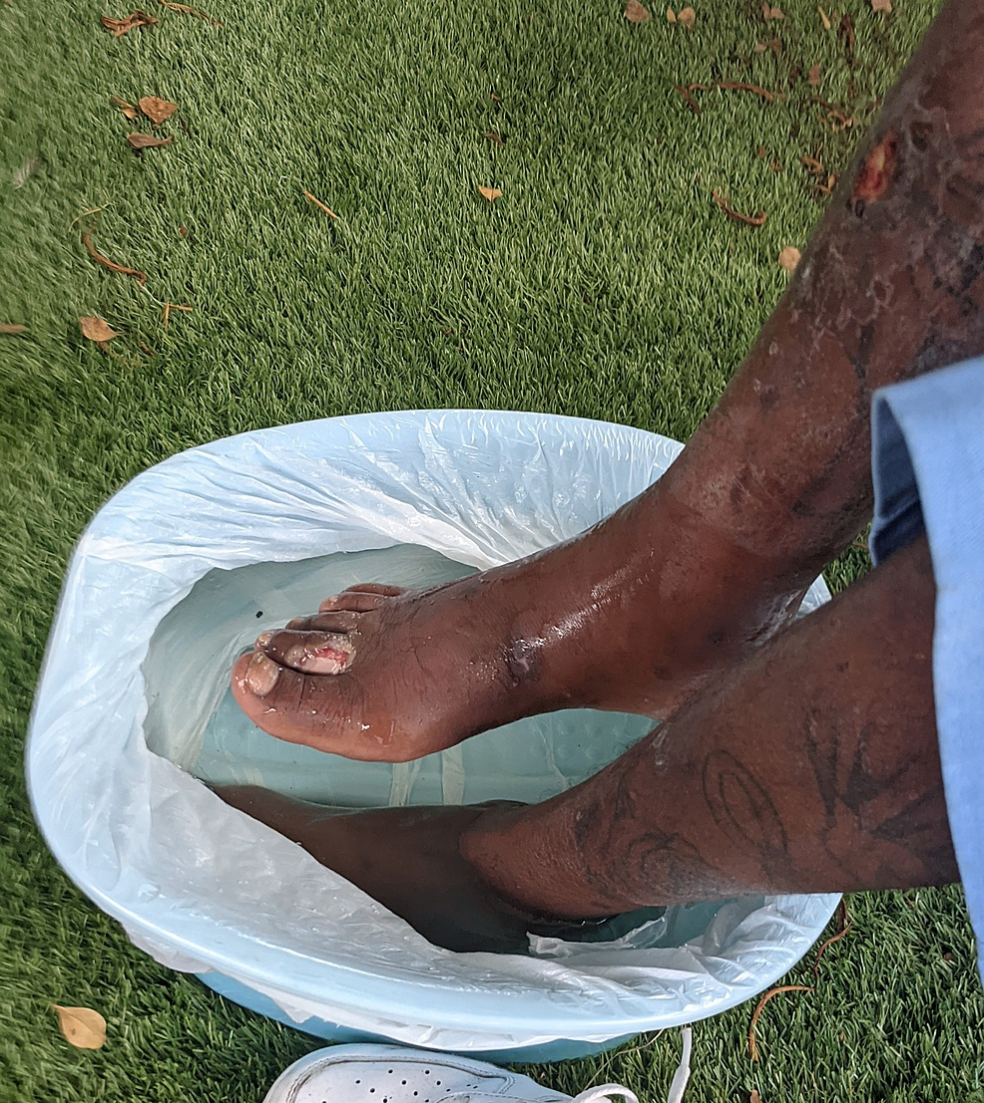
Initial examination of right lower extremity lesions during the foot-washing event. Note the ulcerated right second toe and dark borders around the proximal shin lesion.

**Figure 2:**
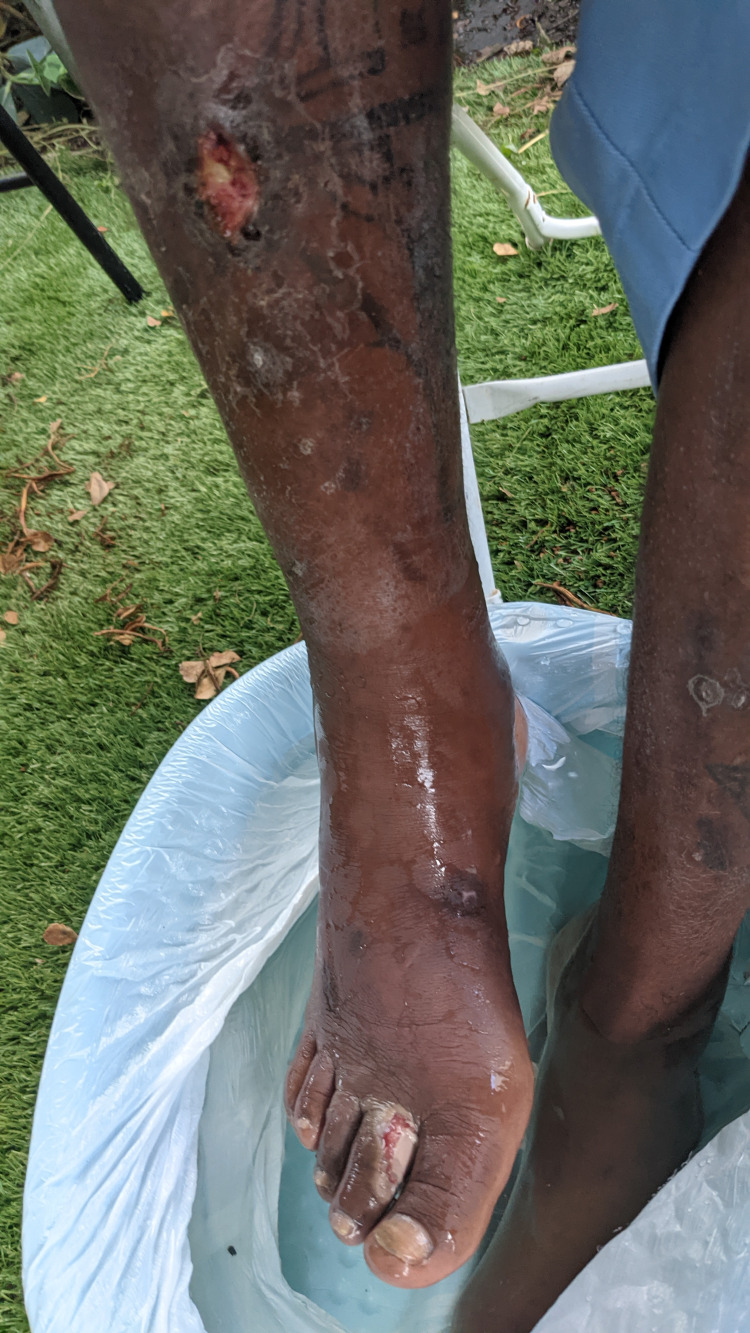
Anterior view of both lesions. The tibial ulceration with peripheral necrotic tissue has central, purulent discharge. The second toe lesion appears to have a separation of the skin, likely as a result of a laceration.

The findings were compelling and generally involved a common theme. Many of the patients who were seen at the foot-washing event had long-standing, untreated lower extremity wounds and lesions. The most notable patient in this cohort was a 41-year-old male with a medical history significant for hypertension, seizures, chronic diarrhea, and cocaine use. He complained of fevers, chills, and some lower extremity swelling. He was noted to have two wounds on his right dorsum and second toe (Figure 1) and one wound on his anterior middle shin (Figure 2). The wounds had pale necrotic margins with surrounding erythema. Upon initial visual examination, the wounds had purulent drainage, and findings were indicative of infection such as warmth, discharge, and systemic fever. The wounds were not tender and were irrigated with sterile saline. He was unsure of the source of his lesions and displayed poor health literacy.

**Figure 3:**
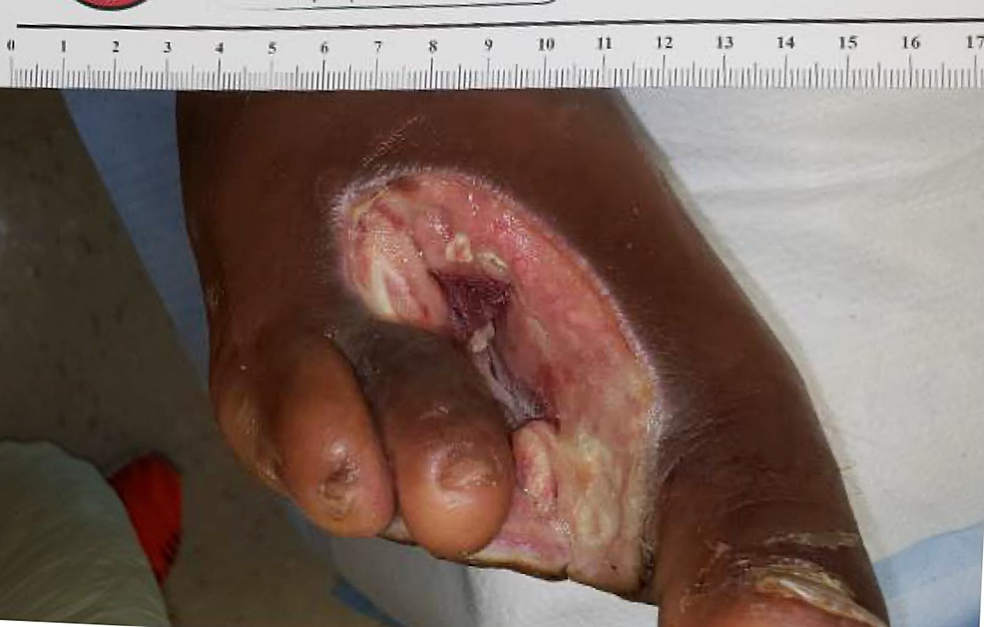
Post-amputation right foot. Due to contiguous spread of infection, the right second and third digits were amputated and the surrounding tissue was debrided.

Further interviewing revealed a history of T1DM, which was poorly controlled due to a lack of access to insulin and reliable healthcare. A point-of-care glucose reading was then measured to be 650 mg/dL. After the administration of 10 units of insulin, the patient was immediately escorted to the ED for further treatment. Upon arrival at the ED, he was immediately admitted to the general medicine floor for DKA management and wound care. Due to the fact that he had multiple underlying acute and chronic health conditions, his hospital course was complicated by multiple infections, and the decision was finally made to amputate his right second and third toes (Figure 3).

## Discussion

Our patient’s story is reflective of the general state of health of many individuals who are unable to access consistent and affordable healthcare. It is likely that earlier outreach and more adequate educational resources would have prevented his deterioration from the complications of DM. In fact, in the United States, diabetics often have higher rates of morbidity and mortality compared to other developed nations [13]. They can frequently find themselves rationing insulin due to a lack of affordability and constantly increasing insulin prices. By some estimates, the price of insulin has increased by approximately 1200% over the past 20 years [14]. This means that individuals who are uninsured or underinsured often face life-and-death decisions daily. This case is not unusual or unforeseeable. Diabetics all across the United States find that the insulin they require to live is far out of reach. This discrepancy is amplified for individuals of lower socioeconomic status, and particularly those who are homeless and unsheltered [15]. Homeless individuals with DM have been found in multiple studies to have higher hemoglobin A1c than age-adjusted diabetics. Preliminary data from the Miami Street Medicine found that among 29 patients tested for blood glucose, at least 52% (N=15) met the criteria for hyperglycemia (blood glucose > 140 mg/dL). Therefore, homelessness appears to be a significant risk factor for uncontrolled DM.

Our patient's skin lesions were suspicious of a systemic process. By eliciting further medical history, it was found that he had long-standing, uncontrolled T1DM. It is likely that his lower extremity lesions developed over the course of at least a few weeks. For many diabetics, the development of skin lesions into wounds is often an insidious process. Even slight trauma or pressure from impaired proprioception can lead to chronic, non-healing wounds and ulcers [16]. Conditions associated with insulin resistance include acanthosis nigricans, acrochordons, and diabetic dermopathy. However, these are usually associated with T2DM. A patient with T1DM is at an increased risk of necrobiosis lipoidica, vitiligo, and bullosis diabeticorum [17]. These conditions are generally rarer than simple diabetic pressure ulcers. Although our patient's lesions are of unspecified origin, they are likely due to a combination of trauma, pressure, or uncontrolled blood glucose.

Through a careful evaluation of our patient’s skin, we were able to evaluate the underlying cause, which in this case was likely of diabetic origin. This is why recognizing the cutaneous manifestations of various diseases is vital when treating persons experiencing homelessness (PEH). Our outreach program was also able to refer other patients with similar lesions but with lower blood glucose levels to community DM health resources.

Our patient was transported to the ED promptly after discovering his elevated blood glucose level of 650 mg/dL. Had he not been taken to the hospital on time, he could have suffered from severe consequences, including sepsis and even death. Unfortunately, his health literacy was also limited, and therefore he was not aware of the importance of taking judicious blood glucose measurements. Additionally, his lower socioeconomic status as an unsheltered homeless individual meant that he did not have regular access to insulin, syringes, and blood glucose monitors. The ulcers noted on his lower extremity displayed signs of infection but were not painful. This suggests that his diabetic neuropathy had progressed to severe levels to the point where he could not feel the pain or infected nature of his wounds. In resource-limited settings such as this case, some of the more common culprits can include vascular insufficiency, DM, and trauma. Point-of-care glucose testing was an invaluable asset in this case, and street medicine teams should generally record blood pressure and blood glucose readings as a standard of care for patients encountered.

The unfortunate conclusion of our patient’s hospital course included the amputation of the right second and third toes. He then stayed in the hospital for approximately one month due to further complications, which included sepsis, amputations, and rehabilitation. Had he been given timely and accessible insulin supplies, his situation likely could have been avoided. It is worth considering whether preemptive treatment and management of DM in unsheltered populations could not only save their lives but also save healthcare costs and ED utilization.

Healthcare providers in low-resource settings and in the absence of extensive testing must rely on the physical examination as well as a thorough evaluation of a patient's skin. This is because underlying diseases can manifest cutaneously and may clue a clinician into a patient’s most concerning issue. Programs similar to the foot-washing event should be regularly conducted in impoverished communities as a means to potentially catch cases similar to ours. Preemptive management of such issues could improve the long-term morbidity of such patients and also reduce the healthcare burden by reducing the likelihood of further complications. Additionally, such events can also serve as an opportunity to improve health literacy of individuals with chronic illnesses who may be unaware of optimal management.

Our patient’s clinical course demands further attention and resource allocation toward preemptive detection of illness and community-based preventive interventions. These upstream efforts require a detailed understanding of current barriers to care and gaps in knowledge. A notable barrier to adequate DM management within the unsheltered population is limited provider understanding of the patient's current living environment. Additionally, community-based studies have demonstrated that peer-guided support groups have had success in promoting health literacy and self-management tactics among diabetic individuals [18]. Programs that engage this vulnerable population could be an effective preventive intervention to identify at-risk patients prior to the insidious and severe decline of mismanaged DM.

## Conclusions

PEH are more severely affected by chronic diseases due to a lack of consistent and appropriate care. Certain illnesses such as DM can have cutaneous manifestations, particularly on the lower extremities. Providers who are treating PEH in resource-limited settings should conduct a thorough physical examination, making sure to note any skin lesions that could be a sign of underlying disease. Additionally, point-of-care glucose testing is a valuable tool in assessing the stability of diabetic patients and determining whether to refer them to the ED. Regular community outreach programs can help identify chronic diseases before the development of severe complications and can be a useful resource for improving patient health literacy.
